# Forecasting Energy Consumption in the EU Residential Sector

**DOI:** 10.3390/ijerph17072259

**Published:** 2020-03-27

**Authors:** Vincenzo Bianco, Annalisa Marchitto, Federico Scarpa, Luca A. Tagliafico

**Affiliations:** Division of Thermal Energy and Environmental Conditioning, University of Genoa—DIME/TEC, Via All’Opera Pia 15/A, 16145 Genova, Italy; annalisa.marchitto@unige.it (A.M.); federico.scarpa@unige.it (F.S.); tgl@ditec.unige.it (L.A.T.)

**Keywords:** energy, forecasting, energy efficiency, residential sector

## Abstract

The present paper aims to introduce a top down methodology for the forecasting of residential energy demand in four European countries, namely Germany, Italy, Spain, and Lithuania. The methodology employed to develop the estimation is based on econometric techniques. In particular, a logarithmic dynamic linear constant relationship of the consumption is proposed. Demand is estimated as a function of a set of explaining variables, namely heating degree days and gross domestic product per capita. The results confirm that the methodology can be applied to the case of Germany, Italy, and Spain, whereas it is not suitable for Lithuania. The analysis of elasticities of the demand with respect to the gross domestic product per capita shows a negative value for Germany, −0.629, and positive values for Italy, 0.837, and Spain, 0.249. The forecasting of consumption shows that Germany and Italy are more sensitive to weather conditions with respect to Spain and an increase in the demand of 8% and 9% is expected in case of cold climatic conditions.

## 1. Introduction

Buildings are supposed to be responsible for the 40% of energy consumption at European Union level [1], therefore, it is of paramount importance to understand which measures can be implemented in order to mitigate their impact on the environment.

In light of these, different countries promoted and implemented a legislation framework aiming at supporting and prescribing the implementations of measures to increase the energy efficiency of buildings [2]. For example, the European Union emanated the Energy Performance Buildings Directive, as well as challenging targets for 2030 and 2050 in terms of increase of energy efficiency, renewable energy generation, and decrease of carbon emissions [3]. Within this complex set of regulations, the buildings sector is considered pivotal, since together with transportation, it is one of the areas with the largest improvement potential.

In order to check the feasibility of these targets as well as to design appropriate compliance strategies, the development of energy demand models is mandatory. Many authors engaged with research devoted to the introduction of models for the forecasting of energy consumption and, usually, two categories can be identified, namely bottom-up and top-down models.

Bottom-up models are based on a detailed description of the energy system of a country, region, etc., and on the basis of the intensity of the developed activities, e.g., industrial processes, transportation, heating, etc., the amount of energy consumed and the corresponding level of emissions are estimated [4]. Usually, these models are data intensive, since a large number of variables are necessary to provide a detailed description of an extended energy system.

Oppositely, top-down models determine the estimation of future energy consumption, or other parameters (e.g., price, generation, etc.), on the basis of the forecasting of relevant macro-variables, such as gross domestic product (GDP), population, climatic conditions, etc., which are linked together by an equation, whose coefficients are estimated on the basis of statistical or other methodologies, e.g., econometric regressions, neural networks, etc. [5,6].

Many examples related to both methodologies are available in the literature and each of the approaches have different advantages and disadvantages, therefore, a general framework that can be applied for all the cases does not exist.

In general, the methodologies to be applied depend on data availability and scope of the analysis to be developed. In terms of accuracy, it is not possible to establish the superiority of an approach with respect to the other, provided that the model is correctly specified, and the input data are consistent.

Bottom-up models are largely utilized to develop energy studies for a specific sector of activity, e.g., power generation [7], transportation [8], etc., or for the implementation of multi-sectoral analyses [9]. The scientific literature is full of examples and case studies related to the application and development of bottom-up energy models and a selection of them is presented in the following.

For example, the building sector attracted the interest of many authors since it offers relevant opportunities to save energy and carbon emissions. To this aim, Bianco et al. [10] modelled electricity and natural gas consumption in the Italian hotel sector by using the LEAP platform, developed by the Stockholm Environment Institute. The model allows understanding of the amount of energy consumption per hotel category and support of energy policy decisions for the implementation of energy efficiency measures.

Similarly, the UK residential buildings sector was analyzed by Kannan and Strachan [11] in order to check how to satisfy the target of 60% reduction of carbon emissions in 2050. They modelled the UK building sector with a detailed bottom-up approach, but also framed it in the broader context of the UK economy. The other sectors interacting with the buildings were modeled in a coarser way. Their model allows identification of the areas of possible action in order to satisfy the given target.

Furthermore, the European building sector was studied by Fotiou et al. [12], who developed a technical-economic bottom-up model representing the European building stock and its future evolution. The scope of the model is to evaluate the different options in order to reach the decarbonization of the sector. On the basis of their model, they conclude that the electrification of the consumption is a reasonable choice to reduce carbon emissions.

Additionally, the power sector attracted the interest of many researchers, because it is one of the main sources of carbon and pollutant emissions, therefore it is of paramount importance to optimize its energy mix in the long term.

To this aim the Chinese power sector was analyzed by Liu et al. [13], in particular they considered the area of Tianjin. The scope of the study was to analyze the impact of different policies on the mitigation of the carbon emissions. For such a reason a bottom-up model of the power sector was developed in the TIMES modelling platform, provided by International Energy Agency, and different energy policy scenarios were simulated and evaluated.

Likewise, the Croatian power system was considered by Prebeg et al. [14], who focused on the diffusion of renewable generation and on the possible integration of electric vehicles. The model takes into account the individual modelling of the thermal power plants representing the Croatian generation fleet and minimizes the global generation cost by considering a set of constraints. The study concludes that it is necessary to have a certain level of thermal power plants in the system due to the variability of renewables.

Finally, Li and Trutnevyte proposed an analysis of the UK power sector [15]. They developed a detailed representation of the power generation and its evolution with the aim to estimate the necessary investment to comply with the 2050 UK policy objectives. They complemented a TIMES modelling approach with a Monte Carlo analysis in order to develop an uncertainty analysis and provide an investment range, rather than a single value, necessary to obtain the given policy target.

Other authors developed models involving more than one sectors, in order to take into account the interdependencies among them. Two examples are given by Pietrapertosa et al. [16] and Amirnekooei et al. [17].

In particular, Pietrapertosa et al. [16] developed a detailed model of the Italian energy system by using the TIMES modelling platform by focusing on the analysis of air pollutants and greenhouse gas (GHG) emissions. They analyzed different policies to mitigate the impact of the emissions.

Whereas, an integrated energy-environmental model was developed by Amirnekooei et al. [17] for Iran. They utilized the LEAP modelling platform for implementing demand side strategies in order to optimize the supply and demand balance for the country. They focused on the savings of fossil fuels, in particular natural gas and crude oil. Their detailed model allowed simulation of the impact of different system management strategies and to quantify the corresponding impacts in terms of energy consumption, pollutants, and carbon emissions.

A comparable variety of studies are developed by using the top-down approach, which is especially employed for the forecasting of long-term energy consumption.

Different methodologies have been employed and applied to a large selection of case studies for typologies and geographic coverage. For example, Sen et al. [18] proposed a model for the forecasting of natural gas in Turkey by utilizing different regression models using a set of socioeconomic indicators as explaining variables. In particular, they found that GDP and inflation rate were the most significant explaining variables. Their study concluded that the natural gas demand will increase in the next years and it is necessary to switch energy consumption from natural gas to other sources of energy in order to guarantee the energy supply.

A study related to the long-term forecasting of the natural gas demand in China is proposed by Ding [19]. He developed a self-adapting grey prediction model, which provides superior performances in terms of volatility prediction with respect to traditional grey models, as demonstrated by the validation on 2002–2014 historical data. 

An improved Holt–Winters exponential smoothing methodology is applied by Jiang et al. [20] to forecast the electricity monthly load for a Chinese city. The classical Holt–Winters (H-W) approach is improved by considering the fruit fly optimization algorithm to select the best smoothing parameter in the H-W equation. The results show that this model substantially improved the prediction accuracy even when a few training data are considered.

Different authors [21,22,23] employed an artificial neural network (ANN) for the forecasting of energy consumption in different countries. In particular, Ekonomou [21] developed an ANN model by employing the multilayer perceptron model (MLP), which allows to test different ANN architectures in order to choose the optimal one. Likewise, Kialashaki and Reisel [22] applied the ANN approach to the prediction of energy demand for the USA industrial sector. The main input of the model is represented by the GDP and the costs of the different energy carriers. Finally, Azadeh et al. [23] applied an ANN-MLP model to the monthly electrical load forecasting in Iran.

Recently Ghoddusi et al. [24] reviewed machine learning methodologies applied to energy economics and finance with particular emphasis on energy prices, demand forecasting, risk strategies, and analysis of the energy trends.

Top down models can be also successfully used for more detailed applications, such as the optimization of energy efficiency interventions [25], real time demand side management [26], and benchmarking strategies for real time energy management [27].

The forecasting of energy demand is becoming a relevant issue, because more and more countries are setting challenging targets in terms of energy savings, therefore, it is paramount to estimate the expected energy demand in order to estimate the volume of investments necessary to comply with the given energy policy targets. Among the different groups of countries, the European Union is very active in defining innovative and challenging energy strategies, as it was the case of the 20-20-20 targets, now updated in the 2030 and 2050 objectives [28,29,30].

As previously mentioned, one of the sectors considered very attractive for its energy efficiency potential is represented by buildings [1,2] and within the building sector residential buildings are of utmost importance. For such reasons it is fundamental to investigate the development of models for the forecasting of the energy demand in the residential building sector 

Despite the relevant importance of the topic, only a few examples are available in the literature. A case is represented by the study proposed by Isaa and van Vuuren [31], which addresses the issue of estimation of residential energy consumption for heating and cooling purposes at global level. Furthermore, the case of Croatia is addressed by Puksec et al. [32], who developed a bottom-up model of the Croatian residential sector. Similarly, Dilaver and Hunt [33] proposed a top-down model to investigate electricity consumption in the Turkish residential sector.

The analysis of the reviewed literature confirms that studies focused on the forecasting of energy consumption in EU buildings are scarce. At the same time, the EU is at the forefront in the promotion of energy efficiency policies, therefore, it is necessary to cover this lack and develop studies for the estimation of energy consumption in the building sectors, especially in a forecasting venue. The present paper aims to bridge this relevant gap.

The scope of the present paper is to develop a top-down model of residential energy demand based on multiple regressions using selected socio-economic indicators as explaining variables. The proposed model will be tested on four European countries, namely Germany, Italy, Spain, and Lithuania. Germany is considered since it is the largest consumer of energy in the EU, Italy and Spain are large consumers as well and with similar features, whereas Lithuania is a small country with a different household stock (i.e., presence of many Soviet buildings in the building stock). The proposed model can be applied to other countries as well.

Once the “business as usual” (BAU) expected energy demand up to 2030 is determined, different scenarios will be built by considering various evolution trends of the explaining variables.

It is supposed that the model and discussion contained in the present manuscript will be useful for policy makers and energy planners.

The paper is structured in the following way: Section 2 reports an analysis of the trend in energy consumption and in relevant variables for the target countries (e.g., Italy, Germany, Spain, and Lithuania), furthermore, the model is introduced, discussed, and validated. Section 3 provides a deep discussion of the obtained results and Section 4 summarizes the conclusion of the present research work.

## 2. Materials and Methods

### 2.1. Data Analysis

To develop the energy forecasting model proposed in the present paper, it is necessary to analyze the trend of fundamental variables determining the energy consumption in the residential sector.

**Figure 1:**
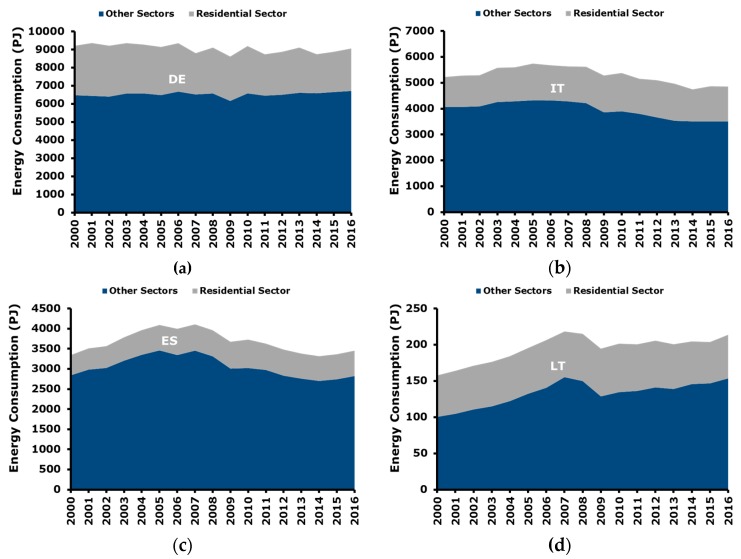
Evolution of energy consumption in the target countries: (**a**) Germany; (**b**) Italy; (**c**) Spain; (**d**) Lithuania. Other sectors group service, industry, transportation, and electricity generation sectors.

Figure 1 summarizes the trends of total and residential energy consumption in the target countries of the study [34], namely Germany, Italy, Spain, and Lithuania.

As for Germany (Figure 1a), it can be said that the evolution of the energy consumption in “other sectors” is quite stable. There is a little decrease only in 2009 which can be ascribed to the global economic crisis which started in that year. On the other hand, in the following years, the crisis effect was already absorbed. As for residential consumption, a more irregular consumption pattern is detected. This is due to the closer correlation with the climatic conditions, which are volatile by definition and determine a large amount of residential energy consumption.

The Italian context is reported in Figure 1b. A relevant decrease of consumption in the other sectors from 2009 onward can be noticed. This can be interpreted as the result of the global economic crisis which started in the period 2008–2009 and has been affecting Italy for approximately the following 10 years. Residential energy consumption is more stable and in analogy to Germany more fluctuations are present in the consumption pattern. As previously said, they are correlated to the volatility of climatic conditions.

The Spanish energy consumption pattern is showed in Figure 1c. The other sectors trend is similar to Italy, with a relevant decrease starting from 2009 and lasting for the following years. On the other hand, differently from in Italy, in the last years (e.g., from 2014 onward) the consumption started to increase again, and this can be interpreted as a sign of improvement of the economic situation. As for the residential energy consumption, the consumption trend appears to be quite regular during the considered time horizon.

Finally, trend of energy consumption in Lithuania is plotted in Figure 1d. A regular increase of energy consumption from 2000 up to 2007 can be noticed, mainly driven by the sectors other than residential. Then, a relevant decrease was registered in the period 2008–2009 and from 2010 onward consumption started to increase again and in 2015–2016 they approximately reached the pre-crisis level.

**Figure 2:**
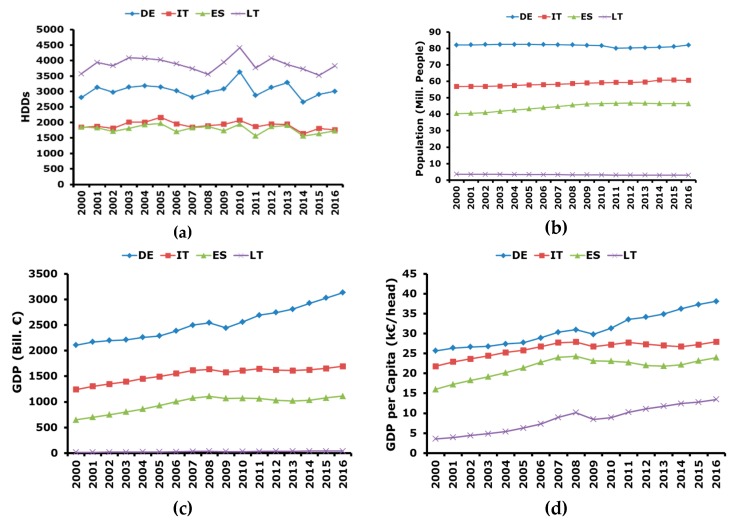
Trend of relevant climatic and macro-economic variables determining energy consumption: (**a**) heating degree days (HDDs); (**b**) population; (**c**) gross domestic product (GDP); (**d**) GDP per capita.

The historical trend of relevant climatic and macro-economic variables is reported in Figure 2. 

In particular, heating degree days (HDDs) [34] (Figure 2a), are considered as an indicator of the country average climatic conditions. Specifically, the higher the HDDs are and the colder the climate is, therefore residential energy consumption can show a certain degree of correlation with HDDs. Figure 2a highlights that Lithuania presents the colder climatic conditions followed by Germany, whereas Spain and Italy are warmer, and they present very similar trends and values of HDDs. It can be observed that also the trend of HDDs in Germany and Lithuania is quite similar, since climatic conditions are not confined to a small area, but they usually involve large territories and Germany, especially the Northern part of the country, and Lithuania are not far from each other.

Population trend is depicted in Figure 2b [34]. It can be immediately noticed that the dimensions of Germany, Italy, and Spain are much larger with respect to Lithuania, which can be considered a very small country with ≈3.5 Mill. inhabitants. The population trend in Italy and Spain show a slight increasing trend in the period 2000–2016. In particular, the trend was increasing till 2008 and quite stable in the following years. As for Germany, the population trend was quite stable from 2000 up to 2010, whereas, in the following years, a slight decrease was registered. A slight increase is registered in year 2016. Probably, these variations are correlated with the global economic downturn.

GDP in nominal values is shown in Figure 2c [34]. It can be seen that, as expected, Lithuania has a much lower GDP with respect to the other considered countries, but with a similar trend. The figure highlights that from 2000 to 2008 a constant growth of the GDP was observed in all the countries. Then, in 2009 there was the global economic crisis and in all the considered countries there was a noticeable reduction of the GDP. On the other hand, after 2009, German GDP continued to increase and in 2010 pre-crisis values were reached. A similar situation happened in Lithuania where in 2012 pre-crisis values were surpassed. A different context is observed in Italy and Spain where there was a stagnation of the economy where pre-crisis values were obtained again in 2015.

It is interesting to notice that the trend in the GDP can be connected with the energy consumption patterns shown in Figure 1. This means that economic development, namely GDP, and energy consumption are still coupled, therefore, the growth of the economy corresponds to an increase of energy consumption. 

Finally, GDP per capita is reported in Figure 2d. The trend is similar to that of the GDP and this is due to the fact that variations in population during the considered time horizon are very limited.

**Figure 3:**
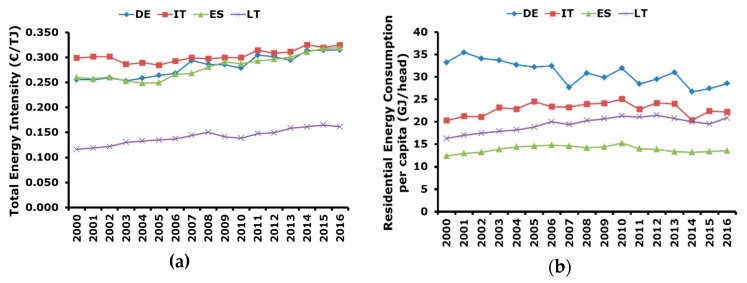
Energy consumption indicators: (**a**) total energy intensity with respect to the GDP (2010 real values); (**b**) residential energy consumption per capita.

Total energy intensity with respect to GDP and residential energy consumption per capita are reported in Figure 3. 

In particular, it can be seen that energy intensities in Germany, Italy, and Spain are very similar from 2008 onward. In the previous years, Italy had the highest energy intensity, whereas Germany and Spain had similar levels. This means that in Germany and Spain energy consumption to generate each unit of GDP grew much more than in Italy. In other words, it can be said that the Italian economy is more energy efficient. Lithuania has a different level of energy intensity, lower than in the other considered countries, but with a clear increasing trend. This can be attributed to the dimension of the country and to the scarce presence of large energy intensive industries.

Finally, the residential energy consumption per capita is presented in Figure 3b and it shows that Germany has the highest value but is characterized by a decreasing trend. The second country in terms of consumption per capita is Italy which shows an increasing trend up to 2010 and then a decrease. This behavior can be probably ascribed to the implementation of energy efficiency polices. Despite the coldest climatic conditions, Lithuania is the third country in terms of residential energy consumption per capita. This can be explained with the differences in the standard of living, which are higher in Germany and Italy with respect to Lithuania and this, independently from the warmer climatic conditions, determines a higher level of consumption due to the presence of more services. Finally, Spain shows the lower residential energy consumption per capita and this can be explained with the milder climatic conditions. Even though in terms of degree days Italy and Spain are similar, it is to be considered that most of the Italian citizens live in the Northern part of the country, where the climate is colder, and this leads to more consumption.

### 2.2. Model Description

To determine the residential energy demand in the selected countries, namely Germany, Italy, Spain, and Lithuania, a top-down model is proposed. The model consists of an equation linking energy demand with a set of explaining variables, which are supposed to determine the consumption.

The mathematical relationship has the following form:

ED = α GDP_PC^β^·HDD^γ^ ED (−1)^ω^, (1)

where ED is the energy demand, GDP_PC is the gross domestic product per capita, HDD represents the heating degree days, and ED (−1) is the one year lagged value of the energy demand. Furthermore α, β, γ, and ω are coefficients to be determined on the basis of the historical data.

Equation (1) is difficult to handle since it is nonlinear, therefore, to simplify the model formulation it is possible to apply a logarithmic transformation in order to have a linear logarithmic function [35].

log (ED) = a + b log (GDP_PC) + c·log (HDD) + d log [ED(−1)],(2)

This form represents a standard constant elasticity function of the consumption, where b and c are the short run elasticities of demand with respect to GDP per capita and HDDs, respectively. From the short run elasticities, it is possible to determine the long run elasticities according to the following equations:

LE_GDP_PC = b/(1−d),(3)

LE_HDD = c/(1−d).(4)

Elasticities are very important indicators, since they provide a measure of the variation of the demand with respect to a change in the values of the explaining variables in the short and long period. Table 1 reports a detailed description of all the considered variables and the corresponding units of measure.

The proposed model does not include energy price as an explaining variable, since residential customers are defined as captive, namely, they do not react to price signals especially in the short run, since they have limited alternatives, therefore, their consumption is not largely affected by the price [36,37,38,39]. Obviously, this is true for limited price increase.

Furthermore the surface of the dwelling stock is not taken into account, since it is dependent on the population (i.e., the floor surface is proportional to the population) as also discussed in [40,41], thus its inclusion may cause multicollinearity problems as population is already embedded in the GDP per capita.

To check for the consistency of Equation (2), one should expect a positive sign for both b and c, as at the increase of the GDP_PC and HDDs it is reasonable to imagine an increase of the demand. An increase in GDP_PC means a higher level of economic activities which usually corresponds to a growth of the consumption due to improved living standard. Higher HDDs relates to colder climatic conditions which determine more energy consumption.

On the other hand, if energy efficiency measures are under development different signs can be obtained. For example, the installation of more efficient devices can turn in a lower energy demand despite a more intensive utilization to guarantee better living conditions.

The estimation of the coefficient in Equation (2) can be obtained by performing a regression based on the ordinary least squares (OLS) methodology. Once the regression is performed, it is necessary to check if the results are statistically significant, therefore, different tests are executed and relevant parameters analyzed, as shown in the following.

Table 2 reports the values of the estimated coefficients for the four considered countries, including fundamental statistical tests, namely t-statistics and F-values, to check for the significance of the obtained values.

The analysis of R^2^ demonstrates that the proposed equation approximates the trend of consumption for Germany, Italy, and Spain quite well, whereas for Lithuania the result is much weaker. The value of the F-statistics confirms that the relationship among the identified variables is not casual for all the considered countries, since it is larger with respect to the critical 99% value of 5.41.

Finally, if the coefficients b and c are considered, namely the short run elasticities with respect to GDP per capita and HDDs, it can be seen that for Germany, Italy, and Spain they exceed the 95% t-value of 2.18 in absolute value. This means that the estimated coefficients are significant.

There is only the exception of the b coefficient for the case of Spain, where 1.651 is less than 2.18. On the other hand, the t-value for a significance of 85% is equal to 1.54, therefore, it can be concluded that the b coefficient has a probability of more than 85% of being significant.

Lithuania represents an exception, since the t-values are much lower with respect to the limit, therefore the estimated equation is not significant, and it cannot be used to estimate residential energy demand in the country. The reason for such a result is to be searched in the structure of the country which is very different with respect to Germany, Italy, and Spain both in terms of country dimension and level of economic activity. Other drivers should probably be used, such as residential buildings surface or degree of urbanization and a different structural equation of the demand needs to be found. Therefore, a more detailed investigation is necessary to develop a forecasting model suitable for Lithuania.

To exclude the presence of multicollinearity, the variance inflation factor (VIF) is calculated [42]. If its value is much less than 10, the absence of multicollinearity can be confirmed. VIF values reported in Table 1 confirm that multicollinearity can be excluded in all the considered cases.

**Figure 4:**
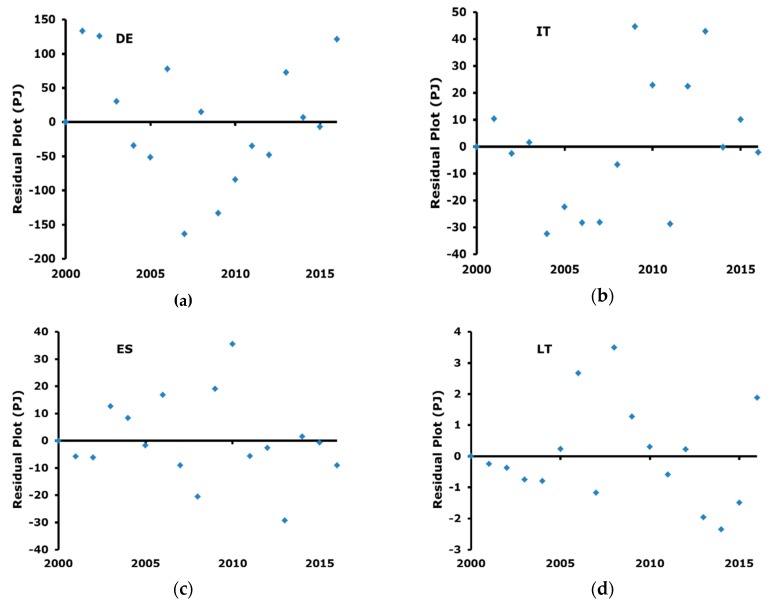
Residual plots for the considered countries: (**a**) Germany; (**b**) Italy; (**c**) Spain; (**d**) Lithuania.

Finally, Figure 4 reports the residual trend. The visual inspection of residuals allow exclusion of some particular trends, e.g., heteroscedasticity and serial correlation, and they can be considered randomly distributed.

In conclusion it can be said that the estimated equation can be considered valid for Germany, Italy, and Spain, whereas for Lithuania it cannot be used, therefore, this country will not be considered in the following. Equation (2) allows determination of the business as usual (BAU) energy demand. Once the BAU trend is obtained, it is possible to determine, on top of it, the impact of possible energy efficiency strategies or possible worsening scenarios depending on variables other than GDP_PC and HDDs. 

### 2.3. Error Analysis and Model Validation

In order to verify the modelling and prediction accuracy of Equation (2) an error analysis is developed. To measure the performance the mean absolute percentage error (MAPE) is taken into account [43]. This indicator can be calculated according to Equation (5):

(5) $$MAPE=\frac{1}{n}\sum_{k=1}^{n}\frac{\left|EC_{E}\left(k\right)-EC\left(k\right)\right|}{EC\left(k\right)},$$

To validate the model, Equation (2) is estimated on data ranging from 2000 up to 2013 and the remaining years are used for comparison purposes, therefore, the coefficients are slightly different from those reported in Table 1. In this way it is possible to analyze the forecasting accuracy of the model on real data. This procedure allows provision of an estimation of the modelling error.

On the basis of the data reported in Table 3, it can be concluded that the proposed model provides reliable estimations of energy consumption in the residential sector of Germany, Italy, and Spain. 

## 3. Results and Discussion

To analyze energy demand in the residential sector of Germany, Italy, Spain, and Lithuania, Equation (2) is proposed and it is supposed that the drivers pushing the demand are climatic conditions, represented by HDDs and the GDP per capita. This relationship is statistically significant for Germany, Italy, and Spain which will be considered in the following. 

Lithuania residential energy consumption cannot be forecasted by Equation (2), since the estimation of the equation coefficients is not statistically significant, therefore, there is a high risk of obtaining spurious results. From the practical point of view, it can be said that the structure of residential consumption in Lithuania is different from Germany, Italy, and Spain. The consumption is driven by other drivers which need to be investigated. For example, the building surface per capita or degree of urbanization rather than the GDP per capita may have a more important role. Nevertheless, future research will be devoted to the development of a dedicated forecasting model for Lithuania.

Table 4 reports the short and long run elasticities of energy consumption with respect to HDDs and GDP per capita for Germany, Italy, and Spain.

As for Germany, it can be noticed that short and long run elasticities of energy consumption with respect to GDP per capita are negative. This means that at the increase of GDP_PC_ corresponds to a decrease in energy consumption. From the economic point of view, it can be said that at the increase of the spending capacity of people, namely GDP_PC_ can be considered as a proxy of the spending capacity, there is a decrease of energy consumption since people invest more and more in energy efficiency measures and devices. The fact that the elasticity with respect to the GDP_PC_ is negative might be ascribed to the saturation of energy consumption in the residential sector. Namely, the current level of consumption is considered satisfactory, which means that each German citizen consumes an amount of energy capable to satisfy all its needs, therefore, there is no necessity to increase. People are also positively reacting to energy policies which encourage and support implementation of energy efficiency measures.

Oppositely, the elasticities with respect to HDDs are positive, which means that the colder the climatic conditions are and the higher the energy consumption is. This is expected, since colder climatic conditions require more energy for building heating.

Different situations are detected in Italy and Spain where the elasticities with respect to GDP_PC_ and HDDs are both positive. Thus, it can be said that the increase of spending capacity encourages consumers to consume more, e.g., by moving into a larger dwelling which requires more energy to be heated, rather than investing in more energy efficient technology as is the case of Germany. This could be due to the fact that climatic conditions in Italy and Spain are much milder with respect to Germany, therefore, the energy bill is less expensive and does not attract the attention of people. To reverse the trend in Italy and Spain it is necessary to promote, more intensively, energy efficiency policies in order to stimulate people to save primary energy. Another possible factor contributing to different elasticities signs may reside in the fact that saturation of energy demand has not yet occurred in Italy and Spain, therefore, there is a residual increase of the demand at the increase of the GDP_PC_.

**Figure 5:**
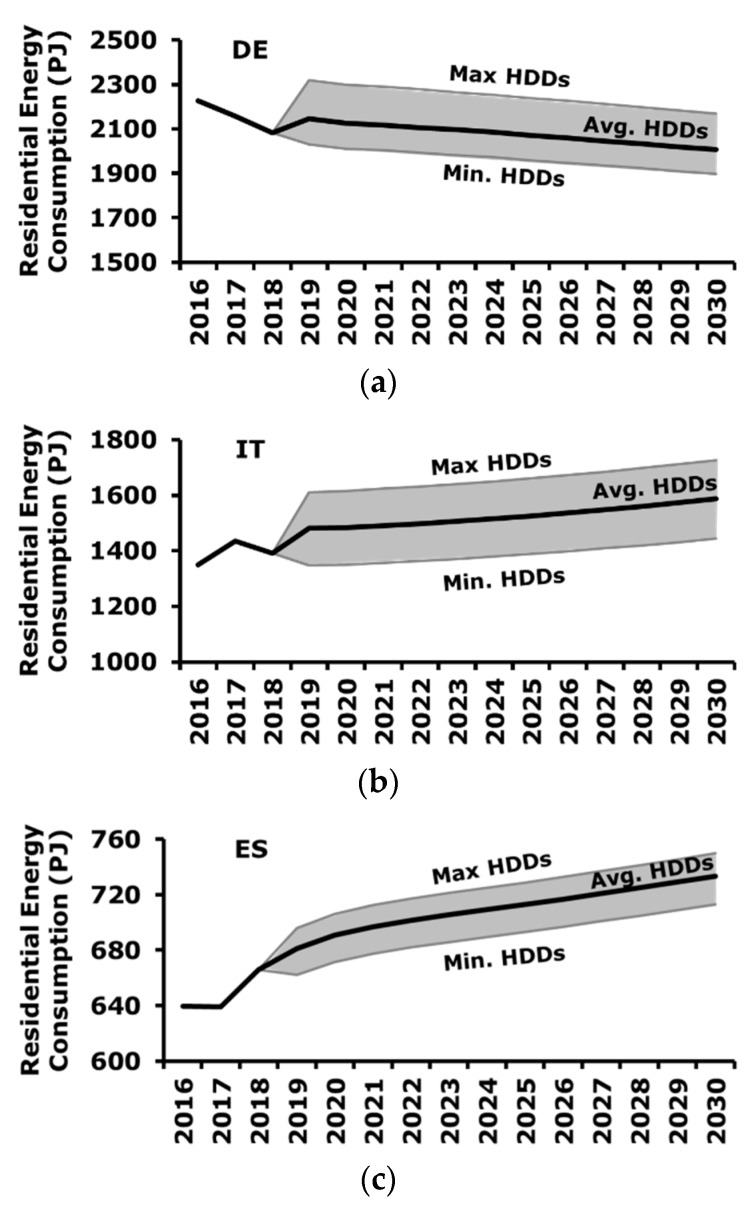
Forecasting of residential energy consumption for the different HDDs scenarios: (**a**) Germany: Min HDDs = 2661, Avg. HDDs = 3028, Max HDDs = 3630; (**b**) Italy: Min HDDs = 1635, Avg. HDDs = 1896, Max HDDs = 2166; (**c**) Spain: Min HDDs = 1563, Avg. HDDs = 1781, Max HDDs = 1972.

The forecasting of residential energy consumption is reported in Figure 5, where three scenarios related to weather conditions are considered, namely average, cold and warm weather conditions. These three scenarios are built by considering average, maximum, and minimum values of HDDs in the period 2000–2016, respectively.

**Figure 6:**
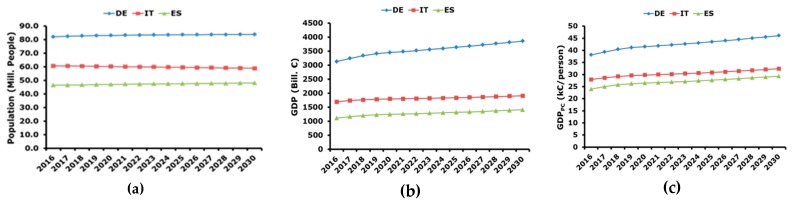
Forecasting of GDP per capita: (**a**) evolution of population; (**b**) GDP nominal values; (**c**) projection of GDP per capita.

Furthermore, the evolution of GDP_PC_ is obtained by considering the projection of population reported in [34] and the outlook of GDP proposed in [44]. Their ratio allows calculation of GDP_PC_ as shown in Figure 6.

Figure 5 highlights that the impact of the climatic conditions, namely HDDs, is relevant especially in Germany and Italy, whereas in Spain the effect is smoother. This is also confirmed by the absolute values of elasticities, which are higher in Germany and Italy with respect to Spain.

As for Germany, rigid weather conditions determine an increase of residential energy consumption of ≈8% with respect to average conditions, whereas warmer weather causes a reduction of the consumption of ≈5%. In the case of Italy, colder and warmer conditions with respect to an average climatic situation provoke an increase and decrease of consumption equal to ≈+/−9%, respectively. Finally, as for Spain, the impact of colder and warmer weather conditions with respect to the average can be estimated in a variation of energy consumption equal to ≈2% and ≈-3%, respectively.

The knowledge of these parameters is relevant for the management of the energy supply, since, according to the expected weather conditions, it is necessary to manage a different level of demand. Furthermore, these considerations support the design of future energy systems, for example in terms of dimensions of energy storage useful to manage peak of the demand.

It is important to mention that the estimation reported in Figure 5 represents the baseline estimation, thus, it considers the projection in the future of the current situation both in terms of policies and users’ behaviors according to the selected explaining variables, but it does not take into account the effect of possible future energy policies or changes in behaviors. The effect of future energy savings actions, in terms of policies or behavioral changes, can be estimated and added on top of the baseline to obtain the final expected energy demand. 

In this regard, the model can be considered very flexible, since it provides the estimation of the baseline which is valid in general and can represent an input for other models where energy efficiency measures are evaluated. An example can be represented by TIMES platform [9], where the baseline demand estimated by using Equation (2) can be given as an input and then the optimal mix of energy efficiency measures to minimize energy consumption can be evaluated.

## 4. Conclusions

The present paper illustrates a top-down methodology for the estimation of energy consumption in the residential sector. An econometric technique was applied to determine the equation representing the energy demand as a function of a set of explaining variables, namely HDDs and GDP per capita. 

The methodology was applied to four European countries, namely Germany, Italy, Spain, and Lithuania. The results demonstrate that the approach is not applicable to Lithuania, probably because of its different dimension as well as a different structure of the consumption.

The analysis of the demand equation for the other countries highlights that in Germany a decrease of the consumption is expected at the increase of GDP per capita, this means that residential customers are willing to invest in energy efficiency measures. The situation is opposite in Italy and Spain. The increase of HDDs, namely more rigid climatic conditions, determines an increase of the consumption in all the countries.

Finally, it can be said that the effect of climatic conditions is more relevant in Germany and Italy, where rigid conditions may lead to an increase of the demand equal to +8% and +9% with respect to average climatic conditions, respectively. 

Furthermore, a downward trend of the demand is estimated for Germany, whereas Italy and Spain show a future increase of the demand.

Future work can be focused on the development of specific models for the forecasting of energy consumption in the Lithuanian residential sector or, more in general, for the Baltic countries residential sector. This area is explored to a very limited extent in the literature, maybe because it is represented by small countries, therefore, further studies could be useful, since the margins for the implementation of energy efficiency measures in the residential sector are very high.

It is the authors’ opinion that the mathematical model and comments reported in the present work may result useful for energy analysts and policy makers in developing future consumption scenarios and energy policies.

## Figures and Tables

**Table 1:** Variables considered in the proposed forecasting model.

Variables	Units	Definition
ED	TJ	Energy demand of the considered country
GDP_PC	k€/head	Country gross domestic product per capita
HDD	°C	Average country heating degree days
LE_GDP_PC	-	Long run elasticities of energy demand with respect to the GDP per capita
LE_HDD	-	Long run elasticities of energy demand with respect to the HDDs

**Table 2:** Regression coefficients for the analyzed countries. Corresponding t-values are reported in parentheses.

Parameter.	Germany	Italy	Spain	Lithuania
a	19.582 (3.309)	1.606 (1.355)	2.433 (1.799)	1.901 (1.034)
b	−0.629 (−3.526)	0.837 (6.158)	0.249 (1.651)	−0.011 (−0.503)
c	0.429 (2.543)	0.638 (8.192)	0.216 (2.260)	0.213 (1.363)
d	−0.121 (−0.516)	−0.058 (−0.520)	0.511 (3.090)	0.678 (3.996)
R^2^	0.85	0.92	0.90	0.68
F	22.6	44.8	34.3	8.4
VIF	0.872	0.958	0.854	0.814

**Table 3:** Validation of Equation (2). The error with respect to the real data is presented in parentheses.

Year	Germany	Italy	Spain
2014	2075 (−4.0%) PJ	1238 (0.1%) PJ	618 (0.4%) PJ
2015	2161 (−3.0%) PJ	1339 (−1.6%) PJ	630 (1.2%) PJ
2016	2150 (−9.1%) PJ	1344 (−0.3%) PJ	647 (2.5%) PJ
MAPE	−5.4%	−0.6%	1.4%

**Table 4:** Elasticities estimation.

Coefficient	Germany	Italy	Spain
b	−0.629	0.837	0.249
c	0.429	0.638	0.216
LE_GDP_PC	−0.561	0.791	0.509
LE_HDD	0.383	0.602	0.442
